# Preface

**DOI:** 10.2174/138920292601241029155110

**Published:** 2024-10-29

**Authors:** Fabio Coppedè

**Affiliations:** 1 Department of Translational Research and of New Surgical and Medical Technologies, University of Pisa, Pisa, Italy

**The Beginning of a New Quarter Century for Current Genomics**

Dear authors and readers, I am pleased to introduce the 26^th^ volume of the journal Current Genomics. The journal has successfully completed 25 volumes, covering a quarter century of research in genome science, and is now ready to continue providing essential reading in the field for the coming years. Indeed, the first issue of volume 26^th^ contains authoritative reviews on biobank regulations in Europe and on pangenome applications, as well as original research articles on geneknockdown methods, molecular evolution, and bioinformatic approaches targeting the genome.

Dear authors and readers, I am pleased to introduce the 26^th^ volume of the journal Current Genomics. The journal has successfully completed 25 volumes, covering a quarter century of research in genome science, and is now ready to continue providing essential reading in the field for the coming years. Indeed, the first issue of volume 26^th^ contains authoritative reviews on biobank regulations in Europe and on pangenome applications, as well as original research articles on geneknockdown methods, molecular evolution, and bioinformatic approaches targeting the genome.

We also invite internationally renowned experts to submit editorials, position papers, and opinion papers that go beyond a simple review of the literature and can make the reader reflect on the real benefits, potential, limits, and ethical implications of genome science.

We extend our gratitude to all of you and our reviewers for helping us maintain the high quality of published articles. We hope that our journal can continue to serve as a benchmark for genome science in the years ahead.

